# Use of a Novel Feeding System to Assess the Survival of a Very Stable Mammalian Virus, Porcine Parvovirus, Within Black Soldier Fly (*Hermetia illucens*) Larvae: A Comparison with Mealworm (*Tenebrio molitor*) Larvae

**DOI:** 10.3390/pathogens13121038

**Published:** 2024-11-25

**Authors:** Antoine Lecocq, Anna Luiza Farias Alencar, Christina M. Lazov, Sheikh M. Rajiuddin, Anette Bøtner, Graham J. Belsham

**Affiliations:** 1 Department of Plant and Environmental Sciences, University of Copenhagen, 1871 Frederiksberg C, Denmark; 2 Department of Veterinary and Animal Sciences, University of Copenhagen, 1870 Frederiksberg C, Denmark; alfal@aqua.dtu.dk (A.L.F.A.); chlv@ssi.dk (C.M.L.); sheikh.rajiuddin@gmail.com (S.M.R.); anettebotner@outlook.com (A.B.); 3 DTU Aqua-National Institute of Aquatic Resources, Technical University of Denmark, 2800 Kongens Lyngby, Denmark; 4 Department of Virus & Microbiological Special Diagnostics, Statens Serum Institut, Artillerivej 5, 2300 Copenhagen S, Denmark

**Keywords:** insect larvae, virus survival, virus ingestion, virus infectivity assays

## Abstract

Insect larvae production offers the potential for large-scale synthesis of high-quality protein that can be used as feed or food. However, currently, there are limitations on the source of substrates for the insect larvae to use. One concern is the potential survival of animal pathogens within insect larvae if their feed is contaminated. In this study, the survival of a very stable virus, porcine parvovirus (PPV), within mealworm (*Tenebrio molitor*) and black soldier fly (BSF) (*Hermetia illucens*) larvae has been analyzed after oral ingestion of the virus. PPV genomic DNA could be readily detected by PCR in both species of larvae up until 9 days post ingestion (DPI), the end of the study period. Furthermore, infection of susceptible PK15 cells by PPV from homogenized mealworm larvae could be detected until at least 3 DPI, using an immunoperoxidase staining method and, up until 9 DPI, with a more sensitive real time PCR assay. Thus, PPV can remain infectious within mealworm larvae during their main growth phase through to their harvesting. However, it may be considered that PPV is exceptional in this respect since it displays unusual stability, e.g., to heat.

## 1. Introduction

There is considerable interest in the industrial scale production of certain insect larvae as a way of converting low-value materials into, for example, useful animal feed. In that regard, mealworm (*Tenebrio molitor*) and black soldier fly (BSF) (*Hermetia illucens)* larvae have been the subjects of considerable attention [1]. However, there are limitations on the nature of the substrates that can be used to feed these larvae. Thus, catering waste products, including animal material, are not currently permitted to be used as feed for the insect larvae within the European Union [2]. A potential hazard associated with the use of waste animal materials is the presence of microbial pathogens, e.g., viruses or bacteria, which could persist within the insect larvae with, or without, their replication.

In earlier studies, we have analyzed the survival, in insect larvae, of two different viruses of pigs, namely porcine respiratory coronavirus (PRCV) [3] and African swine fever virus (ASFV) [4]. The PRCV genomic RNA was only detectable for up to 3 days in mealworm (*T. molitor*) and BSF (*H. illucens*) larvae following exposure to the virus. However, ASFV genomic DNA was detectable (by qPCR) for up to 3 days post exposure in *H. illucens* larvae but for up to 9 days post exposure in *T. molitor* larvae. It is important to note, however, that pigs fed with 50 *T. molitor* larvae, euthanized immediately after feeding on ASFV or 48 h later, did not become infected with this virus [4]. Similarly, pigs fed on 50 *H. illucens* larvae that had been euthanized at 5 h or 24 h after feeding on ASFV-spiked feed did not become infected either. This is despite the fact that each pig received larvae that had ingested up to 10^5.0^ TCID_50_ of ASFV, in total. Thus, it appears that insufficient ASFV was present within the insect larvae to initiate infection by the oral route [4].

Porcine parvovirus (PPV) has a small linear ssDNA genome (about 5 kb in length) that is enclosed within a protein capsid [5]. This virus is known to be exceptionally stable [6,7]. For example, at 5–20 °C, the infectivity of PPV in pig slurry can be maintained for over 40 weeks. This contrasts with the much shorter survival times for other porcine pathogens under these conditions (e.g., swine influenza virus and transmissible gastroenteritis virus (TGEV, a coronavirus related to PRCV) were each fully inactivated after 2 weeks at 20 °C [6]. Thus, parvoviruses may act as a potentially “worst-case” example for virus survival within insect larvae. In this study, we have developed an improved, direct, and controllable system for administering viruses to the *H. illucens* larvae that is more similar to the method used previously with the *T. molitor* larvae. These two feeding systems enabled the uptake and survival of PPV to be readily assessed within these two different types of insect larvae.

## 2. Materials and Methods

### 2.1. Porcine Parvovirus 839

PPV (strain 839 from Denmark, as described (but numbered 893) in [6] and designated as PPV1 839 DNK 1983 in Vereecke et al. [8]), was used for the virus survival studies in Eagles Minimum Essential Medium (EMEM). The virus was grown in primary swine kidney cells in EMEM containing 10% fetal bovine serum (FBS) to a titer of 10^7.3^ TCID_50_/50 µL, which was calculated as described [9]. For determining the titer, the virus was sequentially diluted and used to infect cells in a microtiter plate. PPV-infected wells were identified in an immunoperoxidase test (IPT) by staining with peroxidase-labelled antibodies to PPV essentially as described [10]. In brief, the cells were incubated with the samples, then fixed in ethanol, and incubated with an “in-house” monoclonal antibody towards PPV (LPPV-2). Following washing, the cells were incubated with the secondary peroxidase-conjugated rabbit anti-mouse IgG antibody, substrate (3-amino-9-ethylcarbazole, Sigma-Aldrich, St. Louis, MO, USA) added and the color reaction developed. Wells containing positively stained cells were identified using a light microscope and the titer calculated.

### 2.2. PPV Survival in EMEM

The time required for full inactivation of PPV infectivity at different temperatures in pig slurry and in EMEM have been published previously [6]; for example, at 35 °C, the PPV survived until 21 weeks in pig slurry, and for 14 weeks in EMEM. For the studies presented here, the virus (initial titer 10^6^ TCID_50_/50 µL) was incubated at 5, 20, 35, 40, 45, 50, and 55 °C in EMEM. Samples were collected at pre-selected times and assayed for virus infectivity, using the infected cell staining method, in primary swine kidney cells, as described [6].

### 2.3. PPV 17-8468-1 D #1

PPV isolate: 17-8468-1 D#1 (a PPV strain isolated in Denmark designated as PPV1 8468-1 DNK 2017 in Vereecke et al. [8]) was used for the feeding of insect larvae. It was grown for three days in PK-15 cells (ATCC CCL-33) in EMEM supplemented with 10% FBS, 1% Pen/strep/L-Glutamine (Gibco, Thermo Fisher Scientific, Copenhagen, Denmark) and 1% non-essential amino acids (Gibco). The titer of this PPV stock was 10^4.6^ TCID_50_/_mL_ (determined by titration and staining as described above for PPV 839, except that this titration was performed in PK15 cells, and contained 1.3 × 10^10^ PPV genome copies/mL, as determined by qPCR (see Section 2.12).

### 2.4. Tenebrio molitor (Mealworm) Larvae

The *T. molitor* larvae were sourced from insects reared in the Section for Organismal Biology (SOBI) facility at the University of Copenhagen (UCPH), Denmark. The larvae were housed in plastic containers (16.5 × 10 × 7 cm) with a vented lid. They were kept in the dark at 27 °C and 50–60% relative humidity. For consistency of weight and size, all the larvae used for the assays were 8 ± 1 weeks old and each weighed on average 100 ± 20 mg.

The larvae were fed on ground oats provided ad libitum, and cubes of 1% agar or potatoes were provided as a water source.

### 2.5. Hermetia illucens (Black Soldier Fly) Larvae

The *H. illucens* larvae were obtained from a commercial producer, ENORM (Flemming, Denmark). The larvae were housed in plastic containers (16.5 × 10 × 7 cm) fitted with a lid containing a mesh covered surface and kept in the dark at 27 °C and 50–60% relative humidity. To ensure that larvae would be able to ingest a full aliquot of virus, 8- to 9-day-old larvae were used for the virus exposure studies. The larvae were reared on wet chicken feed (GOLD 4 GALLICO, Versele-Laga pellets in tap water in a ~1:1 ratio (*w/w*), Versele-Laga, Deinze, Belgium).

### 2.6. Feeding of PPV to T. molitor (Mealworm) Larvae

*Tenebrio molitor* larvae were fed with PPV in EMEM, with 10% FBS, essentially as described previously for PRCV [3]; two separate experiments (termed Study 1 and Study 2) were performed using different batches of larvae. PPV (with a level of 6.5 × 10^7^ genome copies/5 μL) was used as the exposure virus. Briefly, *T. molitor* larvae were kept individually for 24 h without access to food or water in plastic medicine cups, subsequently each larva was allowed to consume 5 µL of the virus suspension or virus growth medium (as a negative control). Following exposure (for approximately 5–15 min), each larva and medicine cup were visually inspected to assess if the larvae had consumed all of the provided liquid. Larvae that did not consume the liquid or that had been visibly contaminated on the outside were discarded. The fed larvae were then incubated at 27 °C and 50–60% relative humidity, as above. An Eppendorf tube (2 mL), containing the same PPV suspension used for the insect exposures, was incubated under the same conditions and used as a positive control.

### 2.7. Sampling of T. molitor Larvae

Twenty-five PPV exposed larvae were transferred individually into 2 mL Eppendorf tubes at selected time points (up to 9 days) following exposure. At the same time points, virus samples were collected as positive controls from the Eppendorf tube in the environmental chamber.

The larvae and the virus suspensions samples were then frozen and stored at −80 °C until further processing.

### 2.8. Feeding of PPV to H. illucens (BSF) Larvae

**Figure 1:**
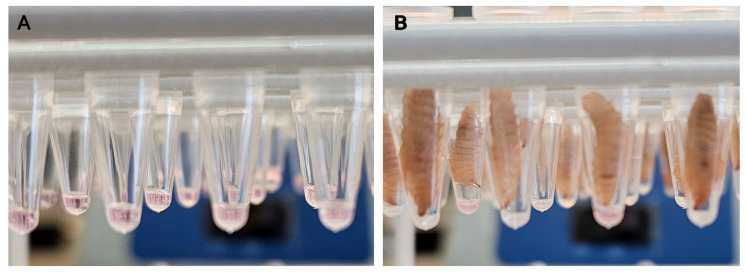
Feeding of black soldier fly larvae with growth medium or virus. (**A**) Aliquots (5 µL) of virus suspension or growth medium, were placed into 0.2 mL PCR Tube Strips. (**B**) BSF larvae were placed with mouthparts facing downwards until they had visibly consumed the liquid within a maximum period of 30 min.

At the start of the experiment, BSF larvae were separated from their feed, rinsed and patted dry on paper towels. The larvae were then placed in a dry container with no feed or water for five hours. During this time, a 5 µL aliquot of virus suspension (treatment) or virus growth medium (control) was placed in open tubes (Eppendorf™ 0.2 mL PCR Tube Strips, VWR, Copenhagen, Denmark). After five hours without feed or water, individual larvae were placed inside one of the tubes, one larva per tube, with their mouthparts facing down towards the 5 µL of liquid (see Figure 1). The larvae were kept in this state for 30 min, under observation, to ensure that they did not crawl back out of the tubes. After 30 min, each larva was taken out of its tube, rinsed and put back in groups into plastic containers, one container per treatment, under the same conditions as described above, and with ad libitum access to wet chicken feed.

### 2.9. Sampling of H. illucens (BSF) Larvae

Twenty-five PPV exposed larvae and ten control larvae were transferred individually into 2 mL Eppendorf tubes at selected time points (up to 9 days) following exposure. At the same time points, virus samples were collected as positive controls from the Eppendorf tube in the environmental chamber. The larvae and the virus suspensions samples were then frozen and stored at −80 °C until further processing.

### 2.10. Processing of Insect Larvae for PPV DNA Detection

After removal from the freezer, each mealworm larva was homogenized individually in 500 µL of EMEM with a 5 mm steel bead (Qiagen, Hilden, Germany) in a Tissuelyser II (Qiagen). Typically, 10 larvae were analyzed for each time point. As a positive control, 5 µL of the PPV stock was added to 500 µL of EMEM and processed in the same way as the larvae. The homogenized larvae were centrifuged at 10,000× *g* for 5 min at 4 °C and 300 µL of the supernatants were collected for DNA purification. The DNA was purified using the IndiMag Pathogen IM48 Cartridge (Indical Bioscience GmbH (Leipzig, Germany, SP947654P608)) in the IndiMag 48 s (Indical Bioscience), according to the manufacturer’s instructions.

The BSF larvae were homogenized similarly, and the DNA was purified using the IndiSpin QIAcube HT Pathogen Kit (Indical Bioscience) in the QIAcube (Qiagen) according to the manufacturer’s instructions. In all cases, the eluted DNA was stored at −20 °C until further use.

### 2.11. Removal of qPCR Inhibitors

The extracted nucleic acids from the mealworm larvae were further purified using a OneStep™ PCR Inhibitor Removal Kit (Zymo Research, Freiburg, Germany, D6030) by centrifugation through Zymo-Spin™ III-HRC Columns, as described by the manufacturer. The samples were stored frozen at −20 °C until further use.

### 2.12. Real Time qPCR Assay for PPV DNA

The qPCR assay, targeting the VP1 coding region, described by Streck et al. [11] was used to detect and quantify the PPV DNA. Briefly, purified samples were assayed usi, ng the RNA UltraSense™ One-Step Quantitative RT-PCR System (Invitrogen, Thermo Fisher Scientific, Copenhagen, Denmark) and the previously described primers (PPV1 FW 5′-CAAGACGATGCACACACACA-3′; PPV1 Rev (5′-TGGTGAGGTTGCTGATTCTG-3′) and probe (6-FAM-CACTAATAGATGCTAACGCATGGG-BHQ1) on a LightCycler^®^ 96 (Roche, Basel, Switzerland) real-time PCR instrument. The thermocycling profile was as follows: 95 °C for 15 min and then a cycle of 95 °C for 30 s, 58 °C for 30 s and 72 °C for 30 s. The FAM dye emission was read during each cycle of the qPCR.

A 200 bp dsDNA fragment, including the primer and probe binding sites, was synthesized as a gBlocks Gene Fragment (by Integrated DNA Technologies, Coralville, IA, USA) corresponding to nt 3095–3294 of the PPV-1 sequence (NADL-2) (GenBank: NC_001718). The fragment was used to generate a standard curve for the qPCR assay (using 10^8^ to 10^1^ copies in 10-fold dilutions, each assayed in duplicate) to enable conversion of Ct values into genome copy numbers.

### 2.13. Processing of Insect Larvae for Infectivity Assays in Cells

As a supplement to the detection of PPV genomes within the larvae, selected *T. molitor* larvae samples from Study 1 on days 0, 3, 6 and 9 post virus ingestion were assayed for the presence of infectious virus in cells. The larvae were homogenized in a TissueLyser II as above, using 1 mL of EMEM 10 × anti (in-house produced medium containing penicillin, amphotericin, neomycin and streptomycin) per larva, at 30 Hz for 5 min and centrifuged at 10,000× *g* for 5 min at room temperature. From each sample, the supernatant was passed through a 0.2 µm filter into a new tube.

### 2.14. PPV Infectivity Assays—By Staining and qPCR

From each sample, undiluted filtrate and 10-fold diluted filtrate in EMEM 10x anti (both with added HEPES buffer) was used for adsorption to cells. Three identical 96-well plates of PK15 cells (100 µL/well with 100,000 cells/mL), seeded the day before, were used for IPT and for the “PCR-check” assay. For this purpose, 50 µL of undiluted filtrate and 10-fold diluted filtrate from each sample were added to cells on each plate. After 1 h of adsorption at 37 °C, the medium was removed from the cells and new pre-warmed EMEM 10 × anti with HEPES was added to all wells. One of the plates was immediately frozen at −80 °C (“PCR check start”), and the other two plates incubated for three days at 37 °C. After this incubation, one of the plates was frozen at −80 °C (“PCR check end”) and the remaining plate was fixed and stained for PPV antigens using the IPT (as described above). Nucleic acids were extracted from 100 µL of each larval filtrate as well as from the harvested cells in medium (“PCR check start” and “-end“, following two freeze-thaw cycles) using the MagNA Pure 96 robot (Roche) with the DNA and Viral NA Small Volume Kit (Roche) and eluted in 50 µL. These samples were assayed for PPV genomes by qPCR, as described above, but using the CFX OPUS 96 (Bio-Rad, Hercules, CA, USA) thermocycler. For evaluation of the “PCR check” sample qPCR results, a minimum reduction of 3 in the Ct value (ca. 8-fold increase) from start to end (after 3 days of incubation) from cells adsorbed with the filtrates was considered to be an indication of PPV infection and thus the presence of infectious virus in the larvae.

### 2.15. Data Presentation

Graphs were prepared using GraphPad Prism 10 (GraphPad Software, Boston, MA, USA).

## 3. Results

### 3.1. A New Method for Administering Virus to BSF Larvae

In previous studies, for exposing the BSF larvae to viruses, it was necessary to add the virus to the dry feed [3,4]. Using this system, it was difficult to control the dose of virus that each larva received, and it was necessary to wash the larvae to remove virus adhering to the outside of each larva prior to nucleic acid extraction. This resulted in a sharp decline, between 0 days post exposure (dpe) and 1 dpe, in the number of larvae in which porcine respiratory coronavirus RNA was detected. In contrast, with the mealworm larvae, it was readily possible to monitor the ingestion of 5 µL aliquots of virus containing medium [3]. Initial attempts to use a similar feeding system for the BSF larvae were unsuccessful as they rapidly died following removal from their feed. A new system has now been developed using BSF larvae as described in Material and Methods. In this system, which more closely resembles the methodology used with mealworm larvae, it is possible to deliver 5 µL aliquots of the virus containing medium to the BSF larvae. While we still observed a decline in the amount of the virus from 1 day post ingestion (DPI), the PPV DNA was detected consistently in the BSF larvae for the duration of the study.

To assess the properties of the new assay system in BSF larvae and the established system for feeding mealworm larvae, we wanted to test the survival of a virus that was known to display high stability, as a test of a potential “worst-case scenario” for the survival of a pathogen within insect larvae.

### 3.2. Thermal Stability of PPV in Eagles MEM

**Figure 2:**
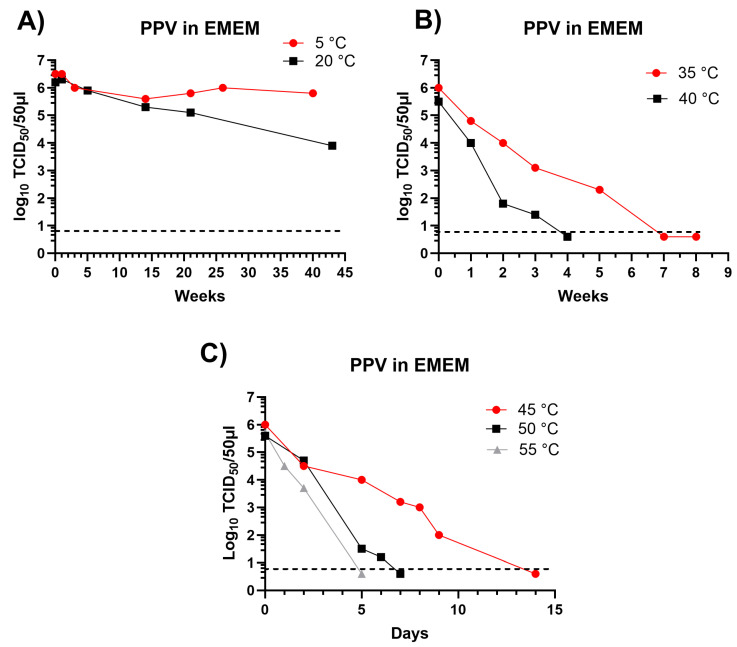
Inactivation of PPV in EMEM incubated at different temperatures. PPV was incubated at 5 °C, 20 °C (**A**), 35 °C, 40 °C (**B**) and 45 °C, 50 °C and 55 °C (**C**) in EMEM for the indicated times and residual infectious virus was assayed by titration in primary swine kidney cells and is presented as log_10_ TCID_50_/50 μL. The minimum level of virus detection in the assays is indicated by the horizontal dashed line. Note that samples were collected at different time points at the various temperatures to cover the relevant periods for virus survival.

PPV has been shown to be remarkably stable when incubated within swine slurry, there was rather little loss of infectivity after 40 weeks incubation at 5 °C or 20 °C and infectivity was still present after 21 weeks at 35 °C [6]. For the studies presented here, the survival of the virus in EMEM was assessed at different temperatures (see Figure 2), a summary of some of the virus survival times in EMEM at different temperatures was presented previously [6] but the time course of the virus survival in EMEM was not provided.

As observed in pig slurry, the PPV retained a high level of infectivity after incubation in EMEM for over 40 weeks at 5 °C or 20 °C (Figure 2A) and residual infectivity was still present after 5 weeks incubation at 35 °C (Figure 2B) and until 5 days or more at 50 °C or 55 °C (Figure 2C). Thus, the high stability of this virus under different conditions has been clearly demonstrated and the results indicated that PPV is a useful agent to test the potential survival of viruses in insect larvae.

### 3.3. Maintenance of PPV in Mealworm Larvae

**Figure 3:**
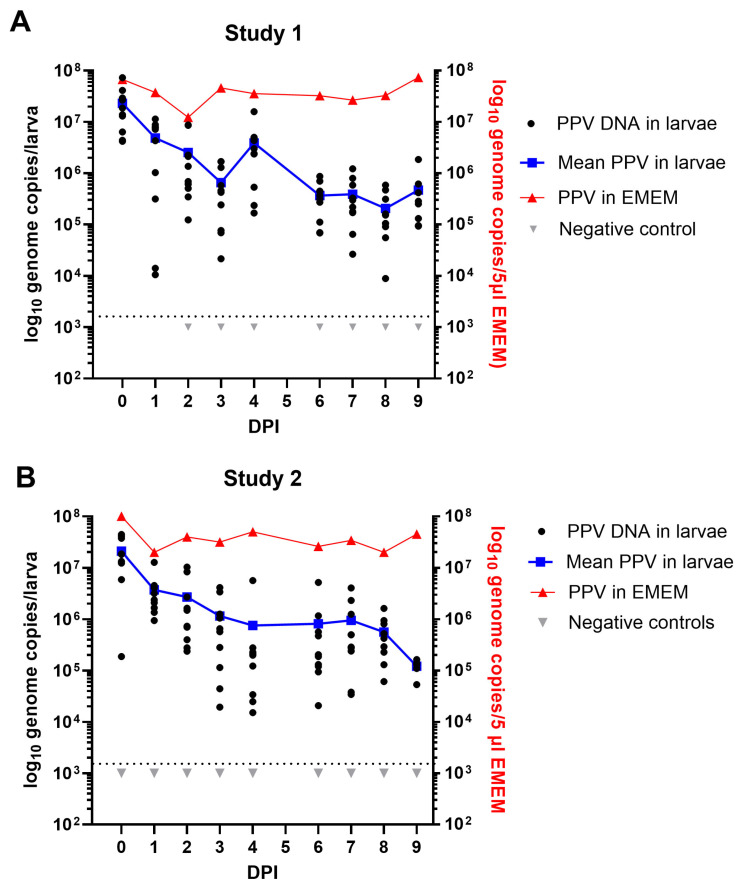
Maintenance of PPV DNA in mealworm (*T. molitor*) larvae. Two separate experiments were performed using different batches of larvae. Typically, 10 larvae were assayed from each time point and the results are plotted individually and as an average. In Study 1 (**A**) and Study 2 (**B**) at 0 days post ingestion (DPI) and subsequently at 1, 2, 3, 4, 6, 7, 8 and 9 DPI the PPV DNA was quantified by qPCR and values were converted to log_10_ genome copy numbers/larva using a standard curve. Levels below 10^3.1^ PPV genomes/larva were below the detection limit (indicated by dashed line). PPV incubated in EMEM at the same temperature (27 °C) was assayed at the same time points.

Following ingestion of 5 µL of EMEM containing PPV (or not, Negative control), the mealworm larvae were incubated for up to 9 days at 27 °C. Results obtained from the extraction and assay for PPV DNA, typically from 10 larvae collected on a daily basis (except for day 5), in two separate experiments using different batches of mealworm larvae, are shown in Figure 3. In both studies, in each 5 µL dose, the larvae will have ingested about 6.5 × 10^7^ PPV genome copies. In Study 1, the extraction and assay for the PPV DNA within these larvae appears to have worked efficiently since, on average, about 2.3 × 10^7^ PPV genome copies/larva were detected on day 0 (Figure 3A). It is apparent that there was then a decline in the level of PPV DNA detected within the larvae during the first few days of incubation, with a mean level of 6.5 × 10^5^ PPV genome copies/larva being detected at 3 days post ingestion. However, during the period of 6–9 days post inoculation there was rather a slow change in the level of PPV in the larvae and they still retained a mean level of about 4.7 × 10^5^ PPV genome copies/larva after 9 days (Figure 3A). This is about 1% of the ingested dose. It is also apparent that the PPV in EMEM remained at an almost unchanged level during the entire 9-day period, confirming its high stability under these conditions (27 °C). Very similar results were observed in Study 2, within an independent experiment, using a different batch of mealworm larvae. The PPV was again found to be present at a level of >1.2 × 10^5^ genomes/larva after 9 days (see Figure 3B).

### 3.4. Maintenance of PPV in BSF Larvae

**Figure 4:**
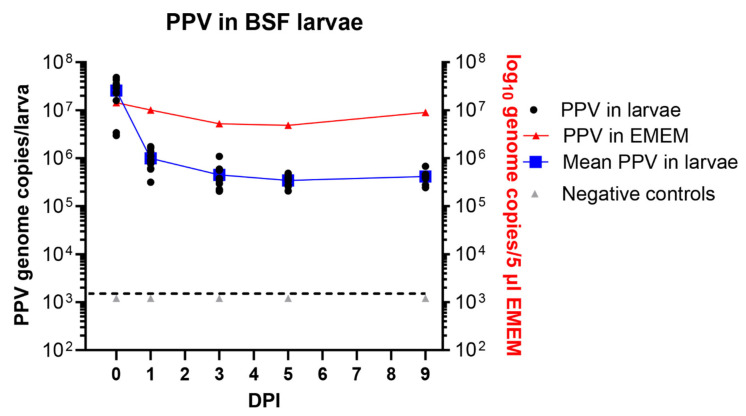
The presence of PPV DNA in individual BSF (*H. illucens*) larvae (typically 10 larvae were assayed at each time point) at 0 days post ingestion (DPI) and subsequently, at the indicated DPI, was quantified by qPCR and values were converted to log_10_ genome copy numbers/larva using a standard curve. Levels below 10^3.1^ PPV genomes/larva were below the detection limit (indicated by the dashed line). PPV incubated in EMEM at the same temperature (27 °C) was assayed at the same time points. Negative controls were larvae fed on EMEM, without virus.

In a similar experiment using BSF larvae, employing the new method for exposure of these larvae to the virus as described above, it was found that the larvae each contained about 2.6 × 10^7^ PPV genome copies on day 0. This again fits well with the expected uptake of about 6.5 × 10^7^ genome copies (Figure 4). There was a marked decline in the level of PPV DNA after 24 h to about 1 × 10^6^ PPV genome copies/larva (on average) and this fell further to 4.5 × 10^5^ PPV genome copies/larva at 3 DPI. However, after this initial decline, the level of viral DNA changed rather little and a mean level of 4.2 × 10^5^ PPV genomes/larva was still present at 9 days post ingestion (Figure 4). This is a very similar level of residual PPV DNA as observed in the mealworm larvae (Figure 3).

### 3.5. Maintenance of PPV Infectivity in Mealworm Larvae

The presence of PPV genomic DNA, which was detectable by qPCR, does not prove the presence of infectious virus. To analyze whether the viral genomes detected within the insect larvae after incubation were present, at least to some extent, within infectious virus particles then selected mealworm homogenates were tested for the presence of infectious PPV. This was achieved using two different methods, firstly by staining for PPV antigens in PK-15 cells and also by running a parallel “qPCR check” assay to determine whether replication of the PPV genome had occurred in the cells.

**Figure 5:**
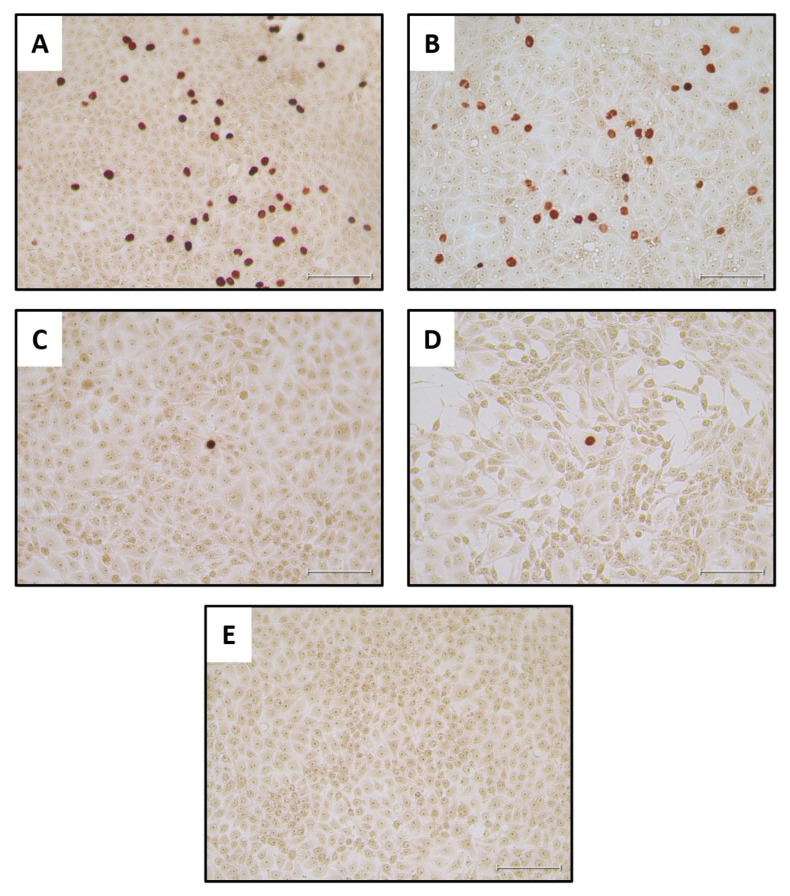
Detection of infectious PPV in PK-15 cells. The cells were treated with the indicated samples, incubated for 72 h and stained for the presence of PPV antigens. (**A**), positive control, PPV virus stock, diluted 1:100; (**B**), positive control virus stock (diluted 1:100) following incubation at 27 °C for 9 days. (**C**), filtered and 10x diluted mealworm homogenate, prepared from a PPV-fed larva frozen on day 0, was added to cells and, after absorption, incubation was continued for 72 h prior to staining. (**D**), filtered and 10x diluted mealworm homogenate, prepared from a larva frozen on day 3 after PPV ingestion, was added to cells and, after absorption, incubation was continued for 72 h prior to staining. (**E**), the cells were treated as for panels C and D, but the mealworm homogenate was derived from a larva that had been fed on EMEM without PPV. A summary of the staining results for each of the larvae tested is shown in Table 1. No staining was detectable from the larvae incubated for 6 or 9 days following feeding on the virus. The scale bar indicates 100 µm.

As a positive control for the IPT, cells were treated with the diluted PPV stock without incubation (Figure 5A) or following its incubation for 9 days at 27 °C (Figure 5B). These results showed that there was no marked reduction in the virus infectivity titer after the 9 days of incubation in EMEM, as expected from Figure 2. Reading the wells with cells that had adsorbed undiluted larval filtrate was challenging. Therefore, it was clearer to observe positive staining results wells when the cells had adsorbed the 10-fold diluted filtrates (see Figure 5C–E) although this obviously reduces the possible number of infected cells observed within each image. The collected staining results showed that infectious PPV could be detected in the mealworm larvae at 0 DPI and up until 3 DPI, when 3 out of the 5 tested virus-fed larval samples stained positive for PPV antigens (see Table 1). However, no staining was observed in cells treated with homogenates produced from larvae at 6 or 9 DPI or in cells treated with homogenates from larvae fed with MEM (Table 1 and Figure 5E).

In an attempt to improve the sensitivity of the assay for PPV infectivity, the growth of the virus was also assessed using a qPCR assay for PPV DNA on the potentially infected cells. Assays were performed on cells that were frozen immediately after virus absorption and also following incubation for 72 h. In this “PCR check” of cells treated with mealworm homogenates, the results showed that all 5 of the virus-fed larvae tested from day 0 and day 3 post PPV ingestion contained replication competent PPV (Table 1). Furthermore, 3 out of 5 larvae from day 6 and 3 out of 5 larvae from day 9 post PPV ingestion also contained PPV that was able to infect the PK-15 cells (Table 1). Note that, in some cases, a better amplification of the PPV was observed using diluted homogenates than with the undiluted samples. It may be that chemical agents present in the mealworm homogenates are detrimental to the cells and hence some of the diluted samples allowed more efficient PPV replication (see Table 1). It is concluded that this ”PCR check” assay for PPV replication was more sensitive at detecting low levels of infectious virus than the immunostaining for infected cells, as may be expected.

## 4. Discussion

As observed previously for PPV in pig slurry [6], it was demonstrated here that PPV retains its infectivity in EMEM for long periods of time (>40 weeks at 5 or 20 °C) and for a significant period (more than 5 days) even at 50 °C (Figure 2). Non-enveloped viruses, such as parvoviruses, are considered to be more resistant to harsh environmental conditions than other viruses and are, therefore, often used to validate disinfection protocols against viruses [12]. In a previous study, PPV was shown to be the most resistant virus to several chemical and physical disinfection methods, highlighting the stability of PPV [13]. Thus, this very stable virus appears to represent a good tool for the assessment of a “worst-case” virus survival within insect larvae that could potentially feed on food waste (although this is currently not permitted by EU regulations).

Using a novel feeding system for BSF larvae and a previously described oral ingestion method for mealworm larvae [3,4], it has been possible to show the efficient uptake of PPV into these two distinct types of larvae (Figure 3 and Figure 4). During an incubation period of up to 9 days, the PPV genome remained readily detectable by qPCR, although only about 1% of the initial levels of virus were still present at the end of the incubation period. Presumably, at least part of the fairly rapid loss of virus that occurred in the first day after virus ingestion resulted from simple excretion of the virus from the gut. However, it was apparent that after this initial decline in virus content, the residual viral genomes were maintained quite efficiently with little change in viral DNA content during the last 3 days of the experiments (Figure 3 and Figure 4). It is not known where the viral DNA is located within the insect larvae. Furthermore, it is not known how long the virus would remain detectable. Further studies would benefit from an in-depth analysis of the presence of the virus within various insect tissues and their frass (excreta) post-ingestion of the virus. However, for the purposes of the current study this information is not important since if the larvae are ingested as feed, then any virus associated within the larvae will be consumed. It was more important to know whether the viral genomes were part of infectious virus, and the results presented here demonstrated that infectious PPV could indeed be detected within the mealworm larvae. Following the addition of mealworm larvae lysates to PK-15 cells and immunostaining it was possible to detect PPV antigens for up to 3 days following ingestion of the virus by the larvae. Furthermore, using a more sensitive, qPCR-based method for the detection of PPV replication, it was possible to show that infectious PPV was present in mealworm larvae for at least 9 days.

This study shows that there was a marked decrease in the presence of virus within larvae within the first few days after ingestion, probably because much of the ingested virus was simply excreted and hence returned to the environment. If the virus is very stable in the environment, as with PPV, then potentially it will still be able to infect susceptible hosts. However, our earlier studies have demonstrated that ASFV, at least, did not have sufficient infectivity by the oral route for pigs following this process [4]. The presence and persistence of viruses of veterinary interest in insects, such as infectious laryngotracheitis virus (ILTV) and infectious bursal disease virus (IBVD), have been reported in *Alphitobius diaperinus* (lesser mealworm), either collected in poultry farms during disease outbreaks in broilers or in experimental infections [14,15], showing that this insect is able to act as a reservoir [14] for these viruses.

The presence of pathogens in larvae after their ingestion will also be influenced by any treatments of the larvae before they are used as feed or food. Clearly, some extraction and post-harvesting methods (e.g., involving change in pH or heating or use of organic solvents) could be very deleterious to any residual infectivity within the larvae [16].

Mealworms and black soldier flies are two of the insect species that have been mass-produced for feed [17]. However, while their industrialization has proved successful over the last decade, their high cost of production has dampened their adoption and sustainability credentials. One of the identified bottlenecks for optimal sustainability is the substrate and/or feed on which these insects are reared [18,19]. Such materials represent the greatest source of potential biological hazards.

To support any change in current legislation, it will clearly be necessary to analyze the survival of other pathogens within insect larvae used for feed production, but the systems used here demonstrate that the necessary tools exist. Efforts to standardize risk assessment methods are already underway [16] and could benefit from these new exposure methods that allow for controlled acute exposure to contaminants in these two different insect species. Furthermore, in cases such as the one presented here, it remains to be determined where the virus is being maintained within the larvae following an initial drop in virus content and what tissues are involved. These methods could also have a role to play in studies directly involved with the health of the insects. Historically, the industrial mass-production of insects has suffered from epidemics caused by various entomopathogens [20]. While new molecular and genomic tools allow fast identification of novel pathogen risks [21,22,23] without the complications of in vivo studies, demonstrating the (re-)infection potential of such agents is more challenging and could benefit from the methods described here. Especially in the case of the black soldier fly larvae, which are considered highly resistant to pathogens [24], but for which no robust exposure method has existed until now.

## Figures and Tables

**Table 1:** Detection of PPV infection in PK15 cells.

Larva	DPI	Replicates Positive, with Maximum ΔCt in Bold	Maximum ΔCt ^1^	qPCR Conclusion	PPV Antigen Staining
L1	0	Undiluted/**Diluted**	15.09	+ ^2^	+ ^2^
L2	0	Undiluted/**Diluted**	13.99	+	+
L3	0	Undiluted/**Diluted**	23.55	+	+
L4	0	Undiluted/**Diluted**	20.52	+	+
L5	0	Undiluted/**Diluted**	16.32	+	+
L9	3	Undiluted/**Diluted**	15.25	+	+
L10	3	Undiluted/**Diluted**	20.21	+	+
L11	3	**Undiluted**	11.07	+	−
L12	3	Undiluted/**Diluted**	20.67	+	+
L13	3	Undiluted/**Diluted**	16.28	+	−
L17	6	**Undiluted**	11.56	+	
L18	6	none	0	−	−
L19	6	none	0	−	−
L20	6	**Diluted**	13.88	+	−
L21	6	**Undiluted**/Diluted	13.41	+	−
L25	9	**Undiluted**	10.51	+	−
L26	9	**Diluted**	12.79	+	−
L27	9	**Undiluted**	13.27	+	−
L28	9	none	0	−	−
L29	9	none	0	−	−
^3^ Pos con 10^−2^	9		6.59	+	+
Pos con 10^−3^	9		7.85	+	+
Pos con 10^−4^	9		6.68	+	+
Pos con 10^−5^	9		−1.62	−	−

Mealworm larvae L6-L8, L14-L16, L22-L24 and L30-L32 were fed MEM as negative controls and harvested on days 0, 3, 6 and 9, respectively, and their homogenates were all negative in the qPCR and PPV antigen staining assays. ^1^: The ΔCt was calculated as the difference between the Ct measured in cells harvested immediately after sample absorption (t = 0 h) and that obtained from cells harvested after incubation for 72 h. Although a threshold of at least a change in Ct of 3 had been preset for a positive result (see Section 2), in practice, there was a reduction of at least 6 in Ct value (ca. 64-fold amplification) in the positive samples. ^2^: + indicates infection detected; − indicates no infection detected. ^3^: Pos con is the positive control virus stock (at indicated dilutions) kept in the insect incubator throughout Study 1 (9 days at 27 °C).

## Data Availability

The original contributions presented in the study are included in the article, further inquiries can be directed to the corresponding authors.
